# Bacterial contamination of pediatric whole blood transfusions in a Kenyan hospital

**DOI:** 10.1111/j.1537-2995.2009.02344.x

**Published:** 2009-12

**Authors:** Oliver Hassall, Kathryn Maitland, Lewa Pole, Salim Mwarumba, Douglas Denje, Kongo Wambua, Brett Lowe, Christopher Parry, Kishor Mandaliya, Imelda Bates

**Affiliations:** From the Centre for Geographic Medicine Research (Coast), Kenya Medical Research Institute/Wellcome Trust Research ProgrammeKilifi, Kenya; the Liverpool School of Tropical Medicine and the Department of Medical Microbiology and Genitourinary Medicine, University of LiverpoolLiverpool, UK; the Department of Paediatrics, Imperial College LondonLondon, UK; and the Coast Provincial General Hospital and Regional Blood Transfusion CentreMombasa, Kenya

## Abstract

**BACKGROUND:**

Hospitalized children in sub-Saharan Africa frequently receive whole blood transfusions for severe anemia. The risk from bacterial contamination of blood for transfusion in sub-Saharan Africa is not known. This study assessed the frequency of bacterial contamination of pediatric whole blood transfusions at a referral hospital in Kenya.

**STUDY DESIGN AND METHODS:**

This was an observational study. Over the course of 1 year, bacteriologic cultures were performed on 434 of the 799 blood packs issued to children by the blood bank of Coast Provincial General Hospital, Mombasa. Clinical outcome was not assessed.

**RESULTS:**

Forty-four bacterial contaminants were isolated from 38 blood packs—an overall contamination frequency of 8.8% (95% confidence interval, 6.1%-11.4%). Sixty-four percent of the bacteria isolated were Gram-negative. Many of the isolates are usually found in the environment and the most likely source of contamination was considered to be the hospital blood bank.

**CONCLUSION:**

Bacterial contamination of whole blood may be a significant but unrecognized hazard of blood transfusion for children in sub-Saharan Africa. Further work is needed to clarify the extent of the problem and its clinical consequences. Increased awareness and adherence to basic principles of asepsis in the hospital blood bank may be important immediate interventions.

Emergency whole blood transfusion for severe anemia is common in hospitals in sub-Saharan Africa particularly for women with pregnancy-related complications and in young children.^1^ Thirteen to 48% of all children admitted to hospital are transfused.^2^–^6^ There is very limited information from the region on the adverse consequences of blood transfusion but what data there are suggests that they are common.^7^,^8^

In sub-Saharan Africa blood donations are usually collected into single bags. Postcollection processing and component preparation are limited and blood for transfusion is usually only available as whole blood in standard volume (approx. 500 mL) units.^8^ The provision of preprepared small volumes for pediatric transfusion is rare and when children are transfused it is common practice for hospital blood banks to draw smaller volumes from standard blood units after cross-match. The original bag is placed back in the refrigerator and the unused blood may be issued to another child (or children, if divided again) until the expiry date of the donation. In this way scarce blood stocks are used more efficiently but the division of blood volumes contained within single collection bags inevitably breaches the integrity of a closed system. Such practices combined with unclean working conditions, warm ambient temperatures (20-35°C), high relative humidity (80%-90%), and unreliable refrigeration may combine to produce a significant hazard of bacterial contamination.

There are no previously published studies relating to the bacterial contamination of blood in sub-Saharan Africa. The objective of this study was to establish the frequency and nature of bacterial contamination in whole blood issued to children receiving a blood transfusion at a health facility in Kenya.

## MATERIALS AND METHODS

The study took place from February 1, 2006, to January 31, 2007, at Coast Provincial General Hospital (CPGH), Mombasa. Blood for transfusion is supplied to the hospital as standard units by the Regional Blood Transfusion Centre and blood is transfused as whole bood. Blood packs issued by the blood bank for transfusion to children aged 14 years or less were eligible for the study. (The term blood “pack” is used here to refer to the variable volume of blood issued to children by the hospital blood bank. This is usually less than a standard “unit” and may or may not be in the original collection bag.) Research ethics committees in Kenya (KEMRI/National Ethics Committee; CPGH Ethics Committee) and the United Kingdom (Liverpool School of Tropical Medicine) approved the study protocol.

Blood packs were sampled at the time of issue from the hospital blood bank to the ward. The blood in the pilot tubing was squeezed into the main pack (with which it is in continuity) three times with a tube stripper to ensure thorough mixing of the blood. The tubing was then disinfected with 70% methanol, clamped twice with a hand sealer at a distance of 10 cm from the pack, and removed from the pack by cutting between the two clips. Both cut ends were washed with 70% methanol and the pack was then issued as usual. In a separate laboratory, pilot tube segments were immersed in 70% methanol for 10 minutes. Using a no-touch technique in a laminar flow hood, 4 mL of blood was aspirated from each pilot tube with a sterile needle and syringe.

Blood samples were inoculated into 40 mL of liquid-phase medium (brain heart infusion with sodium polyanetholsulfonate) and incubated at 35 to 37°C for 7 days. On Days 2 and 7, a smear was made from each sample, Gram-stained, and examined microscopically. At the same time points, all samples were subcultured using standard methods onto blood (7% horse blood), chocolate (5% horse blood), and MacConkey agar plates. Additionally all liquid-phase media were inspected daily and subcultured if signs of bacterial growth were present.

Plates were incubated at 35 to 37°C in air for 48 hours with the blood agar (BA) and chocolate agar (Choc) plates in candle jars. Plates were inspected for bacterial growth at 24 hours (BA, Choc, MacConkey) and 48 hours (BA, Choc). Smears were made from positive plates, Gram-stained, and examined microscopically using standard methods. Standard combinations of biochemical tests were used to confirm the identity of isolates (API, bioMérieux, Durham, NC). All laboratory procedures and equipment were internally quality controlled, and the KEMRI-Wellcome Trust Programme laboratory, which has international quality accreditation, performed regular external quality assessments.

## RESULTS

Over the 12 months of the study, the CPGH blood bank issued 799 blood packs to 798 children and 434 (54%) of these were cultured (Table 1). Packs were sampled and therefore issued for transfusion, a median of 17 days after the blood was donated (range, 0-36 days).

**TABLE 1:** Characteristics of the children transfused and blood packs sampled

Characteristic	All packs issued	Packs sampled
Children aged 14 years or less		
Number	798	434 (54%)
Sex (% male)	59	56
Age (median)	18 months	18 months
Volume of blood requested	300 mL	260 mL
Blood issued		
Days after donation (median)	18	17

Forty-four bacterial contaminants, representing 17 species/genera, were isolated from 38 blood packs—an overall contamination frequency of 8.8% (38/434; 95% confidence interval, 6.1%-11.4%; Table 2). The majority of the organisms isolated were Gram-negative (28/44; 64%). Most of them are usually found in the environment and are not human pathogens or commensals. In two instances, two different contaminants were isolated from the same unit, and on two occasions, three different contaminants were isolated from the same unit.

**TABLE 2:** Organisms isolated by duration of storage at the time of issue (• = 1 isolate)

		Storage time (week from donation)					
Organism and usual habitat		1	2	3	4	5	n
Gram-negative organisms							
*Acinetobacter species*	w, s				•	•	2
*Aeromonas hydrophila*	w		•				1
*Aeromonas sobria*	w					•	1
*Brevundimonas vesicularis*	w, s	•					1
*Burkholderia cepacia*	w, s	•					1
*Enterobacter sakazaki*	w, s	•					1
*Klebsiella pneumoniae*	w, g	•••				•	4
*Ochrobactrum anthropi*	w		•			•	2
*Oligella urethralis*	u					•	1
*Pseudomonas aeruginosa*	w, s	•••					3
*Pseudomonas fluorescens*	w, s		•				1
*Pseudomonas stutzeri*	w, s	••	•				3
*Rhizobium radiobacter*	s			•			1
*Shewanella putrefaciens*	w	•••	•			•	5
Unidentified rod						•	1
Subtotal		14	5	1	1	7	28
Gram-positive organisms							
*Bacillus* species	w, s	••		••	••	••	8
*Micrococcus* species	sk	•		•			2
*Staphylococcus epidermidis*	sk			••	••	••	6
Subtotal		3	0	5	4	4	16
Total		17	5	6	5	11	44

w = water; s = soil; u = urinary tract; g = gastrointestinal tract; sk = skin.

**Fig. 1:**
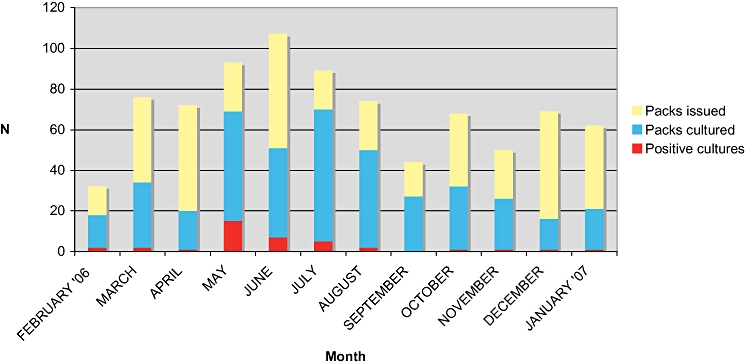
**The number of blood packs issued to children, the number of blood packs cultured, and the number of blood packs from which bacteria were isolated (CPGH, February 2006 to January 2007).**

The number of cross-matches and the number of blood packs issued and sampled varied over time, with the greatest activity from May to August coinciding with the long rains (Fig. 1). The number and proportion of blood packs from which bacteria were isolated also varied over time, with a minimum of zero packs in September (0/27; 0%) and a maximum of 15 packs (15/69; 22%) in May.Twenty-nine (76%) of the 38 contaminated packs were identified during the 4 months from May to August.

## DISCUSSION

The frequency of bacterial contamination of whole blood reported here is more than 2500 times greater than that of red blood cells (RBCs) in industrialized countries (1 in 30,000 units).^9^ The majority of organisms isolated were Gram-negative and most deaths from transfusion-associated bacterial infection in industrialized countries have been attributed to Gram-negative organisms.^10^–^12^ In-hospital case fatality rates reported for children with severe anemia in sub-Saharan Africa range from 8% to 17%,^2^ but in those children who receive a blood transfusion it is not known to what extent transfusion-associated bacterial infection or any other adverse consequences of blood transfusion may contribute to this. Unfavorable outcomes, mostly febrile reactions, have been reported to occur in more than 50% of transfusions at a large hospital in Cameroon—nearly two-thirds of these transfusions were to children.^7^

The most likely source for most of the bacterial contamination observed here was the environment and staff of the hospital blood bank. We suspect this for three reasons: the practice of nonsterile, “open” techniques in the preparation of blood for transfusion; temporal trends over the course of the study; and the nature of the bacteria isolated. These are discussed further below.

The integrity of closed blood packs may be breached on two occasions when blood is prepared for pediatric transfusion at CPGH: first, during compatibility testing, when donor RBCs for cross-match are obtained by cutting off the end of the pilot tubing, expressing a small volume of blood, and then resealing the tubing by tightening a pretied knot; and second, volumes of blood for transfusion, which are less than the standard unit, are often prepared in the manner described in the introduction. Units are divided by draining the anticoagulant from an empty standard single blood collection bag, piercing the pilot tube of a bag containing blood with the needle of the new bag, and expressing the appropriate volume of blood. Tightening a pretied knot above the point of needle insertion reseals the pilot tube of the original bag. Disinfection of the pilot tube, washing hands, and the use of gloves are not routine and a single blood donation may be divided in this manner more than once over the course of its shelf life.

The proportion of blood packs from which bacteria were isolated increased as the demand for blood increased (Fig. 1). This is consistent with contamination from the hospital blood bank environment caused by greater numbers of cross-matches/re–cross-matches and more division/redivision of a limited supply of blood packs. All four packs from which more than one contaminant was isolated were issued in the period May to July. The subsequent steady and then sustained reduction in the frequency of bacterial contamination observed is also consistent with measures introduced by the hospital to try and address the problem. These included increased attention to asepsis through feedback of initial results to laboratory staff and managers (May), increased frequency and thoroughness of cleaning of hospital blood bank (from June), the limited distribution of pediatric blood packs by the Regional Blood Transfusion Centre (August), refurbishment of hospital blood bank laboratory, and installation of air conditioning (December).

In industrialized countries, the organisms most frequently isolated from RBCs are commensal or transient skin commensals from the venipuncture site or pathogens from an undetected donor bacteremia.^10^,^13^–^15^ Gram-positive skin commensals are isolated soon after donation but rarely from stored blood, whereas psychrotropic (cold-tolerating) Gram-negative organisms are not usually detectable until after a period of proliferation during storage.^10^,^13^ The pattern we observed does not support the donor as the most likely source of contamination. First, many of the environmental organisms we cultured are unlikely to have originated from either the skin or the circulation of healthy donors. Second, we isolated Gram-positive skin organisms after 2 weeks of storage and psychrotrophic organisms within 1 week. This suggests a heavy source of contamination from the blood bank environment and/or blood bank technicians near the time of sampling.

### Study limitations

We surveyed the risk of bacterial contamination of blood at a single health facility, which may be unrepresentative. The hospital information systems at CPGH were insufficient to reliably trace blood packs to children who had received transfusions and therefore correlation of suspected bacterial contamination with clinical outcome was not possible. We did not perform anaerobic cultures although strict anaerobes have not been isolated from RBCs in other studies,^10^,^14^,^16^ and the cultures were not quantitative so no judgment can be made about the degree of contamination. The contamination rate we report here could be an underestimate as cultures may not be 100% sensitive if the number of bacteria in the inoculum is low. In addition, further opportunities for bacterial proliferation in warm ambient temperatures may occur on understaffed wards, where blood may wait longer before being administered and/or be transfused over longer periods than recommended.

### Conclusions and recommendations

If widespread, the nature and frequency of bacterial contamination of pediatric whole blood transfusions demonstrated by this study are of considerable public health importance in sub-Saharan Africa. As a matter of priority, further laboratory and clinical surveillance of blood transfusions needs to be undertaken in adults and children to clarify the extent and nature of the problem.

Measures taken by the hospital during the course of the study suggest that awareness of the issue and basic attention to asepsis in the hospital laboratory could have an immediate impact. Low-cost interventions include using a sealed segment of pilot tube to cross-match, using sterile transfer packs to divide units, limiting the number of times a unit can be divided, transfusing divided blood packs within 24 hours, maintaining a clean laboratory environment, and temperature monitoring of fridges. The greater use of multiple-bag collection systems would provide small-volume pediatric transfusion packs requiring no further division, although they are more expensive than single standard volume bags. Hospital clinical and laboratory services should also develop local protocols for the recognition, management, and investigation of transfusion reactions.

Bacteremia is strongly associated with severe anemia in children in sub-Saharan Africa.^2^ Our data suggest that blood transfusion, which is frequently used to treat such children, may also be putting them at risk of bacterial infection. This lends further support to the consideration of antibiotics in the standard management of children with severe anemia.^2^,^17^

In the longer term, we suggest that investment in national blood programs in sub-Saharan Africa can only assure blood safety if there is complementary improvement in hospital laboratory and clinical services. This should be high on the agenda of Ministries of Health and external donors.

## ABBREVIATIONS:

BA: blood agar
Choc: chocolate agar
CPGH: Coast Provincial General Hospital.
